# Sensitivity of a Clinical Decision Rule and Early Computed Tomography in Aneurysmal Subarachnoid Hemorrhage

**DOI:** 10.5811/westjem.2015.7.25894

**Published:** 2015-10-20

**Authors:** Dustin G. Mark, Mamata V. Kene, Natalia Udaltsova, David R. Vinson, Dustin W. Ballard

**Affiliations:** * Kaiser Permanente, Department of Emergency Medicine, Oakland, California; † Kaiser Permanente, Department of Emergency Medicine, San Leandro, California; ‡ Kaiser Permanente Northern California, Division of Research, Oakland, California; § Kaiser Permanente, Department of Emergency Medicine, Roseville, California; ¶ Kaiser Permanente, Department of Emergency Medicine, San Rafael, California

## Abstract

**Introduction:**

Application of a clinical decision rule for subarachnoid hemorrhage, in combination with cranial computed tomography (CT) performed within six hours of ictus (early cranial CT), may be able to reasonably exclude a diagnosis of aneurysmal subarachnoid hemorrhage (aSAH). This study’s objective was to examine the sensitivity of both early cranial CT and a previously validated clinical decision rule among emergency department (ED) patients with aSAH and a normal mental status.

**Methods:**

Patients were evaluated in the 21 EDs of an integrated health delivery system between January 2007 and June 2013. We identified by chart review a retrospective cohort of patients diagnosed with aSAH in the setting of a normal mental status and performance of early cranial CT. Variables comprising the SAH clinical decision rule (age ≥40, presence of neck pain or stiffness, headache onset with exertion, loss of consciousness at headache onset) were abstracted from the chart and assessed for inter-rater reliability.

**Results:**

One hundred fifty-five patients with aSAH met study inclusion criteria. The sensitivity of early cranial CT was 95.5% (95% CI [90.9–98.2]). The sensitivity of the SAH clinical decision rule was also 95.5% (95% CI [90.9–98.2]). Since all false negative cases for each diagnostic modality were mutually independent, the combined use of both early cranial CT and the clinical decision rule improved sensitivity to 100% (95% CI [97.6–100.0]).

**Conclusion:**

Neither early cranial CT nor the SAH clinical decision rule demonstrated ideal sensitivity for aSAH in this retrospective cohort. However, the combination of both strategies might optimize sensitivity for this life-threatening disease.

## INTRODUCTION

### Background

Approximately 80% of non-traumatic cases of subarachnoid hemorrhage are attributable to ruptured cerebral aneurysms, for which delays in definitive aneurysm treatment can increase the risk of disability or death. 1, 2 While the vast majority of aneurysmal subarachnoid hemorrhage (aSAH) cases are identified by cranial computed tomography (CT), the sensitivity of CT diminishes with time such that lumbar puncture is recommended as the definitive test to exclude a diagnosis of “CT-negative” SAH. While two large studies have reported that early cranial CT (i.e. performed within six hours of headache onset) may be up to 100% sensitive for SAH among patients presenting with a normal mental status, 3–6 a prior study by our research group demonstrated imperfect sensitivity of this definition of early cranial CT in the non-academic emergency department (ED) setting. 7 Accordingly, we proposed that sequential application of a validated SAH clinical decision rule (absence of all the following: age ≥40, neck pain or stiffness, headache onset with exertion, loss of consciousness at headache onset) in such a clinical scenario might further reduce the posterior probability of CT-negative SAH to an acceptable level of risk. 8–10

### Goals of this investigation

We sought to further examine the potential incremental gain in sensitivity when applying a previously validated SAH clinical decision rule to a cohort of patients diagnosed with aSAH after presenting to the ED with normal mental status and undergoing early cranial CT.

## METHODS

### Study Population

We screened electronic health records of patients treated within the Kaiser Permanente Northern California (KPNC) integrated healthcare delivery system between January 2007 and June 2013 for case inclusion if they had an ED or hospital encounter with an associated International Statistical Classification of Diseases and Related Health Problems, ninth edition (ICD-9) diagnosis code of SAH (430). Emergency care within KPNC is provided through 21 non-academic medical center-based EDs, serving a population of over 3.3 million Kaiser Foundation Health Plan (KFHP) members. This study was part of a larger project examining outcomes following misdiagnosis of aSAH. Patients were electronically excluded if they had an ICD-9 coded diagnosis of head or neck trauma within 24 hours of the index encounter, lacked continuous KFHP membership within the two weeks preceding diagnosis, were under 18 years of age or had a prior diagnosis of SAH between 2002 and 2006. Data on age, sex and race were electronically collected. We then manually reviewed charts for the following inclusion criteria: initial diagnosis at a KPNC ED, Hunt-Hess clinical grade of 1 or 2 at the time of ED presentation, non-contrast cranial CT imaging within six hours of headache onset, either evidence of SAH on non-contrast cranial CT or greater than five red blood cells per microliter on cerebrospinal fluid analysis, and angiographic evidence of cerebral aneurysm thought to be consistent with the clinical presentation and pattern of hemorrhage visualized on imaging, if applicable. The study was approved by the Kaiser Foundation Research Institute Institutional Review Board with a waiver of the requirement for informed consent.

### Methods and Measurements

Two investigators (DGM and MVK) conducted a structured explicit chart review and abstraction of records using a standardized paper form as part of a larger study examining outcomes following misdiagnosis of aSAH. 11 Abstractors confirmed the inclusion criteria and the final radiologist interpretation of the initial cranial CT, the location and size of the culprit aneurysm and documentation of the presence or absence of the following variables: neck pain or stiffness, loss of consciousness, physical exertion at the time of headache onset, need for external ventricular drainage and treatment of vasospasm during hospitalization. A best modified Rankin Scale (mRS) score at one year was assigned by reviewing neurosurgical, rehabilitation services and primary care clinical notes following hospital discharge, if applicable, using previously validated methodology. 12 We considered a mRS score ≤2 a favorable neurologic outcome. Both abstractors reviewed 20% of the sample to establish the inter-rater reliability of the following variables with an estimated error margin less than 20%: early cranial CT (inclusion criteria), Hunt-Hess grade at ED presentation and the clinical decision rule. 13 All CT examinations were performed without contrast using multi-slice cine technology (16 slice or higher). Either general radiologists or neuroradiologists made the final interpretation of CT images.

### Outcomes and Analysis

The primary outcome of interest was the combined sensitivity of early cranial CT and the SAH clinical decision rule (a negative result for the latter being defined as absence of all four clinical criteria). Secondary outcomes were the independent sensitivities of early cranial CT and the SAH clinical decision rule. Missing variables from the SAH clinical decision rule were imputed as being absent to provide the most conservative estimate of sensitivity. We calculated binomial confidence intervals (CI) using the Clopper-Pearson (exact) method. All statistical analyses were performed using STATA v 13.0 (College Station, TX).

## RESULTS

### Characteristics of study subjects

We identified 155 patients following application of exclusion and inclusion criteria (Figure). The median age was 55 years and 79% were female. Hunt-Hess grade was 2 in 95% of patients, though none of these had notation of a cranial nerve deficit upon initial ED evaluation. The most common aneurysm location was the anterior communicating artery (30%), followed by the posterior communicating artery (21%). Eighty percent of patients had a favorable neurologic outcome one year from initial hospitalization. Summary statistics of the study population are provided in Table 1.

### Main results

Early cranial CT was reported as positive for SAH in 148 patients, yielding an estimated sensitivity of 95.5% (95% CI [90.9–98.2]). The SAH clinical decision rule was likewise positive in 148 patients with the same estimated sensitivity (95.5%, 95% CI [90.0–98.2]). Since the false negative cases for early cranial CT were mutually independent from the false negative cases by the SAH clinical decision rule, the combined estimated sensitivity for application of both early cranial CT and the SAH clinical decision rule was 100% (95% CI [97.6–100.0]). Seven patients (4.5%) underwent lumbar puncture, all of whom had negative early cranial CT interpretations. Pertinent details for false negative cases of early cranial CT and the SAH decision rule are provided in Tables 2 and 3, respectively.

The inter-rater agreement for electronic health record abstraction was 100% for early cranial CT, 87% for Hunt-Hess grade (1 versus 2) and 100% for a negative result on the overall SAH clinical decision rule (71% for neck pain or stiffness in isolation).

## DISCUSSION

The goal of this study was to help clinicians further refine and understand current testing strategies for aSAH, specifically by highlighting the potential gain in sensitivity obtained with the “post-hoc” application of a SAH clinical decision rule following early cranial CT. While we recognize that this was not the original derivation or validation setting of the SAH clinical decision rule, we feel our study demonstrates that such an approach can potentially help inform shared decision-making between clinicians and patients when faced with uncertainty over the absolute sensitivity of early cranial CT, especially in the face of non-low pretest probability.

This testing strategy is similar in concept to performing a serum d-dimer assay to reliably exclude a lower extremity deep vein thrombosis in a patient with a high pre-test probability for disease but a negative lower extremity ultrasound examination; only the combined sensitivities of the two tests offer a low enough post-test probability to forgo further testing. 14 Additionally, although early cranial CT failed to detect aSAH in seven out of 155 cases (4.5%) in our retrospective cohort, this is only a point estimate and is specific to our practice setting. The findings of 100% sensitivity in the prospective Perry et al. 4 cohort and the retrospective Backes et al. 3 study rightfully prompt consideration of using early CT alone to rule out aSAH in those particular practice settings.

Important contextual differences between our study and prior reports involve both spectrum bias and radiologist staffing practices. Perry et al. 4 enrolled patients with acute headaches reaching maximal intensity within one hour. Backes et al. 3 retrospectively identified patients presenting to a SAH referral center with a 50% incidence of SAH among patients undergoing early cranial CT (as opposed to 13% in the Perry et al. study). Thus it is possible that our study cohort includes patients with less severe presentations of aSAH who may be less likely to manifest positive CT findings on early cranial CT. Likewise, radiology staffing at a tertiary neurosurgical referral center as in Backes et al. 3 is not representative of the vast majority of EDs. While the Perry et al. 4 study setting was similar to ours in that radiographic studies were interpreted by a mix of neuroradiologists and general radiologists who routinely interpreted cranial CTs, several of the medical centers participating in that study had active radiology residency training programs with over-reading of studies by faculty in the daytime, making it difficult to extrapolate that level of scrutiny to practicing radiologists in a non-academic hospital setting. Of note, one early cranial CT in that series was initially misinterpreted as being negative by a radiology trainee, and was only retrospectively re-interpreted as positive when a magnetic resonance angiogram performed several days later revealed an aneurysm. This example highlights the potential for introducing hindsight bias by using final written radiology reports as the gold standard for CT interpretations, an issue for all studies on this topic to date.

Finally, it is notable that all of the patients with false negative cranial CT studies met the age criteria of the SAH clinical decision rule. It is thus possible that a post-imaging rule could be further refined for improved specificity and ease of applicability.

## LIMITATIONS

Given the retrospective nature of the study, appropriate characterization of early cranial CT is only as accurate as the available documentation. While the inter-rater agreement for abstraction of this dichotomous variable was 100%, we cannot exclude errors in reporting the actual time of headache onset such that some patients may have been both included and excluded inappropriately. However, such errors in reporting can occur in prospective observational studies as well, and thus is a more general limitation of using historical factors as part of any decision rule.

The completeness of our case identification was also limited by the accuracy of the diagnostic coding from hospital and ED encounters. However, we searched databases specific for diagnostic codes assigned during treatment within KPNC as well as those used to track services billed for outside of KPNC, thus making it unlikely that we failed to capture cases of aSAH that were transferred to a non-KPNC hospital. Regardless, it seems improbable that we failed to capture enough cases of aSAH to appreciably alter our results; for example, to raise the sensitivity point estimate for detection of aSAH by early cranial CT to 99.0%, we would require an additional 545 cases of aSAH, all with positive early cranial CT findings (693/700=0.99).

Given that we conservatively imputed missing variables for the SAH decision rule as being absent, it is also possible that the SAH clinical decision rule may have performed better than reported if missing variables were in fact present. However, recent prospective and retrospective validations of the same SAH clinical decision rule also revealed suboptimal sensitivities at 98.5% and 96.6%, respectively. 8, 15 Additionally, we cannot comment on the specificity of the SAH clinical decision rule when applied following early cranial CT, given the case-only cohort design of our study. It is possible that such an approach would not reduce overall testing rates from current practice, although individual clinicians may be differentially influenced given variable testing thresholds. 16

Finally, we did not include potentially missed cases of aSAH who may have undergone early cranial CT imaging with no confirmatory (LP) or subsequent testing, only to later present with evidence of aneurysm rupture. Inclusion of such cases in this cohort would bias towards a lower sensitivity of early cranial CT since it is impossible to be certain that evidence of SAH would have been otherwise detected (i.e., by lumbar puncture) at the initial evaluation.

## CONCLUSION

In summary, we found the sensitivity of early cranial CT for aSAH to be 95.5% among patients presenting to non-academic EDs in an integrated healthcare system. Application of a SAH clinical decision rule in addition to early cranial CT improved sensitivity to 100% (95% CI [97.6–100.0]). Prospective decision rule refinement and validation of this approach is warranted.

## Figures and Tables

**Figure:**
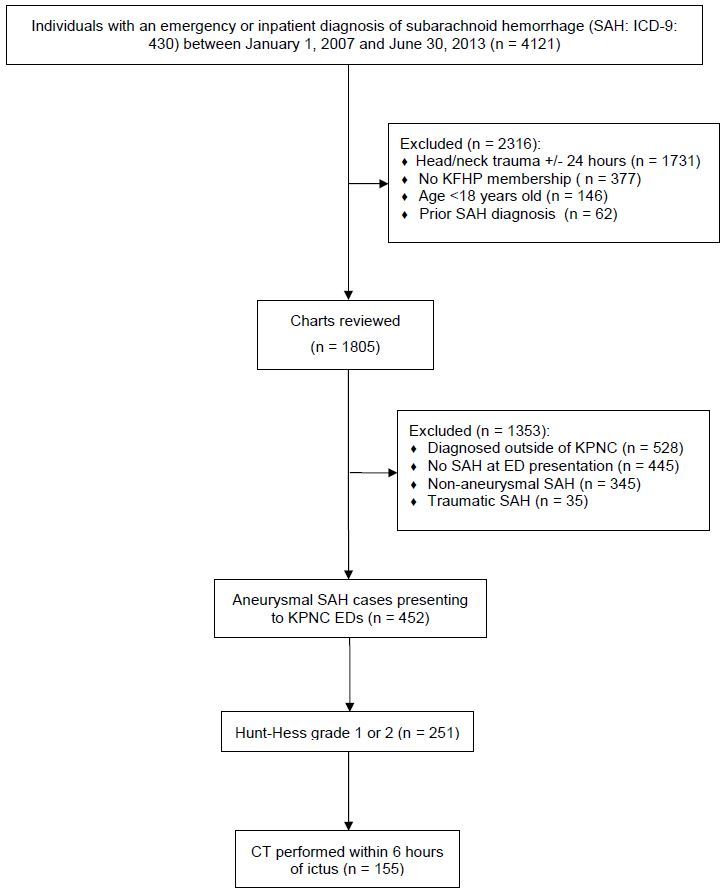
Aneurysmal subarachnoid hemorrhage cohort assembly. *CT,* computed tomography; *ED,* emergency department; *ICD-9,* International Classification of Diseases, Ninth Revision, Clinical Modification (ICD-9-CM); *KFHP*, Kaiser Foundation Health Plan; *KPNC*, Kaiser Permanente Northern California; *SAH*, subarachnoid hemorrhage

**Table 1:** Patient characteristics and outcomes (n=155).

Variable	Value
Age (median, years)	55
Female (%)	79
Race (%)	
Caucasian	42
Black	17
Asian	23
Hispanic	2
Unknown/other	16
Hunt-Hess grade (%)	
1	5
2	95
Neck pain (%)	
Yes	45
Unknown	10
Loss of consciousness (%)	
Yes	14
Unknown	1
Headache onset with exertion (%)	
Yes	15
Unknown	16
Aneurysm location (%)	
ACOM	30
PCOM	21
MCA	15
ACA	6
ICA	8
PICA	5
Basilar	5
Other*	7
Unknown	3
Inpatient treatments (%, n)	
Vasospasm requiring intervention	21 (33/151)
Hydrocephalus requiring EVD	26 (40/154)
Neurologic outcome by one year (%, n)	
Alive	85 (132/155)
mRS ≤2	80 (122/152)

*ACOM*, anterior communicating artery; *PCOM*, posterior communicating artery; *MCA*, middle cerebral artery; *ACA*, anterior cerebral artery; *ICA,* internal carotid artery; *PICA*, posterior inferior cerebellar artery; *EVD*, external ventricular drainage; *mRS*, modified Rankin scale.

* Other locations (n) included the vertebral artery (5), superior cerebellar artery (3), pericallosal artery (3), anterior choriodal artery (3), ophthalmic artery (2) and the superior hypophyseal artery (1).

**Table 2:** Imaging and laboratory details for the seven false negative cranial computed tomography studies.

Age	CT scanner	CT slice thickness	CSF RBCs/microliter**	Xanthochromia	Angiography results
≥90	unavailable	5mm	280000	Yes	5mm ACOM aneurysm
76	GE lightspeed VCT (64 slice)	5mm	517500	No	4mm right PCOM aneurysm
67	GE lightspeed VCT (64 slice)	5mm	408000	No	6mm left PCOM aneurysm
45	GE lightspeed VCT (64 slice)	5 mm	190000	No	4mm left ICA aneurysm
53	GE lightspeed Pro 16 (16 slice)	5mm	49750	No	2mm right PCOM aneurysm
50	GE lightspeed VCT (64 slice)	1.25mm	9960	No	10mm ACOM aneurysm
70	GE lightspeed VCT (64 slice)	5mm	55000	Yes	2mm right vertebral artery

Seven patients presenting with aneurysmal SAH had cranial CT studies performed within six hours of headache onset that were initially reported as negative for evidence of subarachnoid hemorrhage. Diagnosis of SAH was made by lumbar puncture in each case. Details of the CT technology used as well as the results of diagnostic lumbar punctures and formal cerebral angiography are presented for each case.

** CSF RBC counts were the lowest values reported in cases where multiple tubes were analyzed.

*GE*, General Electric; *VCT,* volume computed tomography; *CT*, computed tomography; *ACOM*, anterior communicating artery; *CSF*, cerebrospinal fluid; *RBC,* red blood cell; *ICA*, internal carotid artery; *PCOM*, posterior communicating artery; *SAH*, subarachnoid hemorrhage

**Table 3:** Imaging and clinical details for the seven patients with false negative clinical decision rules.

Age	Neck pain or stiffness	Onset with exertion	Loss of consciousness	CT results	Angiography findings
32	No	Unknown	No	Positive	5mm right ACA aneurysm
39	No	No	No	Positive	6mm left MCA aneurysm
27	No	No	No	Positive	7mm left ACOM aneurysm
32	No	Unknown	No	Positive	6mm right PCOM aneurysm
39	Unknown	No	No	Positive	ACOM aneurysm (unknown size/location)
25	No	No	No	Positive	2mm right ICA aneurysm
29	Unknown	No	No	Positive	Right MCA aneurysm (unknown size)

Seven patients presenting with aneurysmal SAH had false negative results using the clinical decision rule (age >40, presence of neck pain or stiffness, headache onset with exertion, loss of consciousness at headache onset) for subarachnoid hemorrhage. Diagnosis of SAH was made by computed tomography in each case. Details of the decision rule elements and formal cerebral angiography are presented for each case.

*ACA*, anterior cerebral artery; *ACOM*, anterior communicating artery; *CT*, computed tomography; *ICA*, internal carotid artery; *MCA*, middle cerebral artery; *PCOM*, posterior communicating artery; *SAH*, subarachnoid hemorrhage
